# Long-term exposure to PM_2.5_-bound metals and female breast cancer incidence in New Mexico, USA

**DOI:** 10.1007/s11869-026-02079-1

**Published:** 2026-08-25

**Authors:** Yanhong Huang, Charles L. Wiggins, Angela L. W. Meisner, Yan Lin, Li Luo, Xi Gong

**Affiliations:** 1 Department of Biobehavioral Health, The Pennsylvania State University State College, University Park, PA 16802 USA; 2 University of New Mexico Comprehensive Cancer Center, University of New Mexico, Albuquerque, NM 87131 USA; 3 New Mexico Tumor Registry, University of New Mexico, Albuquerque, NM 87131 USA; 4 Department of Internal Medicine, University of New Mexico, Albuquerque, NM 87131 USA; 5 Social Science Research Institute (SSRI), The Pennsylvania State University, University Park, PA 16802 USA; 6 Department of Geography, The Pennsylvania State University, University Park, PA 16802 USA; 7 Institute for Computational and Data Sciences (ICDS), The Pennsylvania State University, University Park, PA 16802 USA

**Keywords:** PM_2.5_-bound metals, Female breast cancer, Incidence, GIS, Environmental health, Exposure assessment

## Abstract

Female breast cancer (FBC) is the most commonly diagnosed malignant cancer and the second leading cancer cause of death among women in the United States. Environmental exposures, particularly PM_2.5_-bound metals, have been increasingly associated with breast cancer incidence. We evaluated associations between long-term residential exposure to PM_2.5_-bound metals and age-adjusted FBC incidence rate at the census tract level in New Mexico from 1997 to 2019. FBC records were obtained from the New Mexico Tumor Registry. Annual metal exposure intensities for chromium, cobalt, copper, manganese, and mercury were estimated using a calibrated Emission Weighted Proximity Model (EWPM) over a 10-year pre-diagnosis window. Generalized Estimating Equations (GEE) with Gaussian estimation was used to estimate rate ratio (RR) for the association between PM_2.5_-bound metal exposure and FBC incidence in New Mexico. Exposure to PM_2.5_-bound chromium (adjusted RR 1.07, 95% CI: 1.05, 1.08), cobalt (adjusted RR 1.08, 95% CI: 1.05, 1.11), copper (adjusted RR 1.06, 95% CI: 1.04, 1.07), manganese (adjusted RR 1.05, 95% CI: 1.02, 1.08), and mercury (adjusted RR 1.05, 95% CI: 1.03, 1.07) show significant associations with increased FBC incidence rate. When exposure was categorized as unexposed, low, medium, and high groups, a significant increasing trend was observed across all five metals. Long-term exposure to multiple PM_2.5_-bound metals is associated with elevated FBC incidence rate in New Mexico. These findings highlight the need for further research and to biologically validate the observed associations and clarify potential mechanisms linking PM_2.5_-bound metal exposure to FBC incidence risk.

## Introduction

Female breast cancer (FBC) is the most commonly diagnosed malignant cancer and the second leading cancer cause of death among women in the United States (U.S.CDC 2024). In New Mexico, breast cancer remains a significant public health concern, with an age-adjusted incidence rate of approximately 116.3 per 100,000 females between 2017 and 2021(NIH 2025). Despite improvements in early detection and treatment, substantial disparities persist in breast cancer incidence and outcomes by geographic location, socioeconomic status, and race/ethnicity (Yedjou et al. 2019) – New Mexico represents one such setting characterized by large rural populations and medically underserved communities.

Breast cancer incidence is influenced by a combination of genetic, hormonal, lifestyle, and environmental risk factors (German et al. 2023; Kaminska et al. 2015; Nouges 1994; Tamimi et al. 2012). Emerging evidence suggests that environmental exposures, particularly to airborne pollutants, may increase breast cancer incidence risk (Wu et al. 2024). Research has investigated the potential links between exposure to criteria air pollutants (CAPs) (particulate matter [PM_2.5_ and PM_10_], nitrogen dioxide (NO_2_), sulfur dioxide (SO_2_), ozone (O_3_), and carbon monoxide (CO)) and breast cancer incidence. Long-term exposure to air pollutants such as NO_2_ and PM_2.5_ showed significant positive associations with increased breast cancer incidence, particularly among postmenopausal women (Wang et al. 2025). Across nine USA states/regions (1973–2007), higher exposures to CO, PM_10_, SO_2_, and nitrogen oxides (NOₓ) were linked to increased FBC incidence risk (Wei et al. 2012). Similarly, it has been found strong temporal and spatial correlations between NO_x_ emissions, motor vehicle density, and breast cancer incidence trends across the USA during the same period (1973–2007) (Chen and Bina 2012). Additionally, the emissions of Polycyclic Aromatic Hydrocarbons (PAHs) and PM_2.5_ were significantly linked to higher breast cancer incidence risk in metro Atlanta compared to rural Georgia in the USA between 1992 and 2011 (Parikh and Wei 2016). Exposure to NO_x_ and PM_2.5_ has also been associated with increased FBC incidence risk in the USA between 1995 and 2007 (Kayyal-Tarabeia et al. 2024). Furthermore, a study in Ontario, Canada, found that long-term exposure to PM_2.5_ and NO₂ have positive associations with breast cancer incidence risk from 2001 to 2015 (Bai et al. 2020). In Los Angeles, California, breast cancer risk was significantly higher among women living within 500 m of major roads, particularly in areas with elevated levels of NOx, NO₂, PM_10_, and PM_2.5_ during the period from 1993 to 1996 (Cheng et al. 2020).

Compared with the extensive studies on CAPs, evidence linking PM_2.5_-bound metals to FBC incidence remain limited, though growing. Emerging studies suggest that exposure to PM_2.5_-bound metals may contribute to cancer development through mechanisms such as generating oxidative stress, DNA damage, and interfere with cellular signaling pathways (Yan et al. 2024). Several metals commonly found in PM_2.5_–such as arsenic, cadmium, chromium, and nickel–are classified as known or probable human carcinogens by the International Agency for Research on Cancer (de Conti et al. 2025; IRAC 2025). These metals can bind to fine PM, enabling inhalation and systemic absorption, potentially initiating or promoting carcinogenesis in hormone-sensitive tissues such as the breast (Nemmar et al. 2013). Epidemiological studies have linked long-term exposure to ambient PM_2.5_-bound metals with elevated risks of lung, bladder, and breast cancer, suggesting a possible role in both initiation and promotion of carcinogenesis (Byrne et al. 2013; Lequy et al. 2023; Liu et al. 2022). In particular, cadmium has been associated with estrogenic activity and has been detected in breast tissue, raising concerns about its role in breast cancer etiology (Filippini et al. 2020; Tarhonska et al. 2022) Women residing in ecologically polluted areas showed elevated concentrations of certain microelements, including chromium, in breast tumor tissue, suggesting a potential link between environmental metal exposure and breast cancer incidence risk (Batyrova et al. 2022). Soil chromium pollution has been reported to be positively associated with breast cancer–related hospitalizations in Southeast China (Song et al. 2023a). In addition, it has been found that women exposed to higher levels of airborne cobalt had increased odds of developing estrogen receptor–negative/progesterone receptor–negative (ER/PR-) breast cancer in Chicago (Kresovich et al. 2019). Similarly, PM_2.5_-bound cobalt exposure was associated with increased breast cancer incidence in a multi-city cohort study in the USA (White et al. 2016). Furthermore, it has been found that mercury bioaccumulation in breast cancer tissues was linked to higher tumor risk (Servadei et al. 2025). High serum nickel levels were significantly associated with increasing breast cancer incidence, suggesting that nickel exposure may be a potential risk factor (Yu and Zhang 2017).

However, evidence on PM_2.5_-bound metals and FBC incidence risk remain limited, particularly in analyses using long-run cancer registry data or examining geographically and demographically diverse populations. To address these gaps, this study investigates the associations between long-term exposure to five PM_2.5_-bound metals (chromium, cobalt, copper, manganese, and mercury) from industrial emission and age-adjusted FBC incidence rate at the census tract level in New Mexico from 1997 to 2019. The five metals were selected because they are commonly emitted from industrial sources, persistent in fine PM that contribute to long term inhalation exposure, as well as the emerging toxicological evidence with FBC (Ali et al. 2024).

## Data and methods

### Female breast cancer data

The New Mexico Tumor Registry (NMTR) provides high-quality cancer surveillance data to support scientific research and cancer control initiatives (NMTR 2024). Cancer site and morphology were coded according to the *International Classification of Diseases for Oncology*, Third Edition (*ICD-O-3*) (Fritz et al. 2000, 2014). We collected 29,719 incident cases of female breast cancer diagnosed among New Mexico residents between January 1, 1997, and December 31, 2019. Cases without geocoded residential coordinates (2.11%) were excluded from the analysis. After applying this exclusion, a final sample of 29,086 FBC incident cases remained for analysis. This study calculated the age-adjusted FBC incidence rate for each of the 499 census tracts in each year based on the age distribution of the female population, using the 2000 U.S. standard population for age standardization (Klein and Schoenborn 2000).

### Industrial air pollution emission and air quality monitoring data

**Fig. 1:**
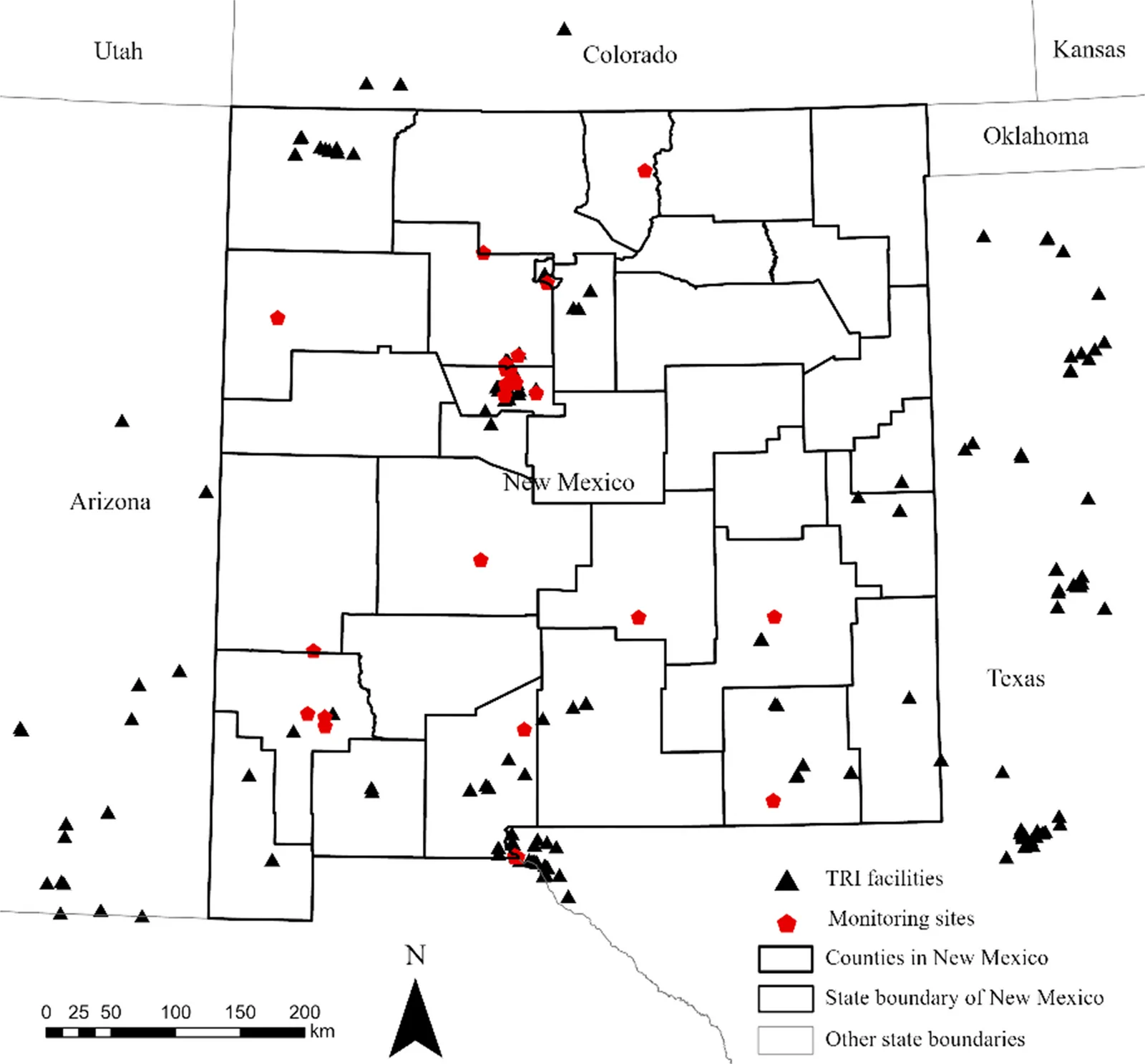
Geographic distribution of metal emission sources and air monitoring sites in New Mexico and its surrounding areas during 1987–2019

The U.S. Environmental Protection Agency’s (EPA) Toxics Release Inventory (TRI) requires industrial facilities to report annual chemical emissions, including types, quantities, and release locations (U.S. EPA 1995). For this study, we used TRI data from 1987 to 2019 for industrial facilities located in New Mexico and neighboring areas of Texas, Colorado, and Arizona, where a total of 199 facilities reported emissions of 18 different metals (Fig. 1). Annual emissions were calculated by combining stack and fugitive releases. Air quality monitoring data were also collected from the EPA’s Air Quality System (AQS) DataMart, which compiles national ambient monitoring results (U.S. EPA 2020). In New Mexico, 28 monitoring sites measured 41 metals over this period, and we used annual mean concentrations to correspond to the emission data (Fig. 1).

### Air pollution exposure assessment

We assessed residential exposure to industrial air pollutants using a calibrated Emission Weighted Proximity Model (EWPM) (Gong et al. 2016a). The model estimates pollutant intensity by combining emission rate, duration, and distance from each source, while excluding emissions beyond a pollutant-specific “effective distance.” These effective distances were calibrated only for pollutants with both emission and monitoring data by choosing the cutoff that maximized the significant Spearman correlation between modeled and observed concentrations. For each PM_2.5_-bound metal, the calibrated EWPM was applied to estimate average annual exposures at each residence for the 10 years preceding diagnosis (Gong et al. 2016b, 2023; Gong et al. 2018a, b; Gong et al. 2018a, b; Zou et al. 2009).

### Identification of potential risk factors

We analyzed the association between residential exposure to PM_2.5_-bound metals and age-adjusted FBC incidence rates across New Mexico census tracts, 1997–2019, using generalized estimating equations (GEE). The GEE approach was chosen to account for potential spatial clustering and repeated measures within census tracts over time. We calculated age-adjusted FBC incidence rates for each census tract (U.S. CDC 2024). We fit Gaussian GEE models to the log-transformed outcome, specifying the dependent variable as $\mathrm{log}$*(age-adjusted*$\;FBC\;incidence$* rate*$\;(per\;\mathrm{100,1000}\;females)+C)$ , where *C* is a small constant (0.00001) added to handle zero rates. The models incorporated year-specific exposure and cancer incidence rate data to evaluate associations.

Each industrial air pollutant was evaluated in a single-pollutant model, where exposure was categorized as unexposed (0) versus exposed (> 0) based on the annual estimated assessment for each chemical. All models adjusted for tract-level covariates: race/ethnicity composition (Non-Hispanic White, Hispanic, Non-Hispanic Black, Non-Hispanic American Indian/Alaska Native [AIAN], Non-Hispanic Asian/Pacific Islander [API], and Non-Hispanic Others (U.S. Census Bureau 2025), female education attainment, median female income, and urbanicity (urban vs. rural) (USDA 2025). This study used the 2010 U.S. census tract boundaries as the geographic unit of analysis. For interpretability, effect estimates can be exponentiated to be called rate ratio (RR), because *C* was small relative to typical rates.

## Results

**Fig. 2:**
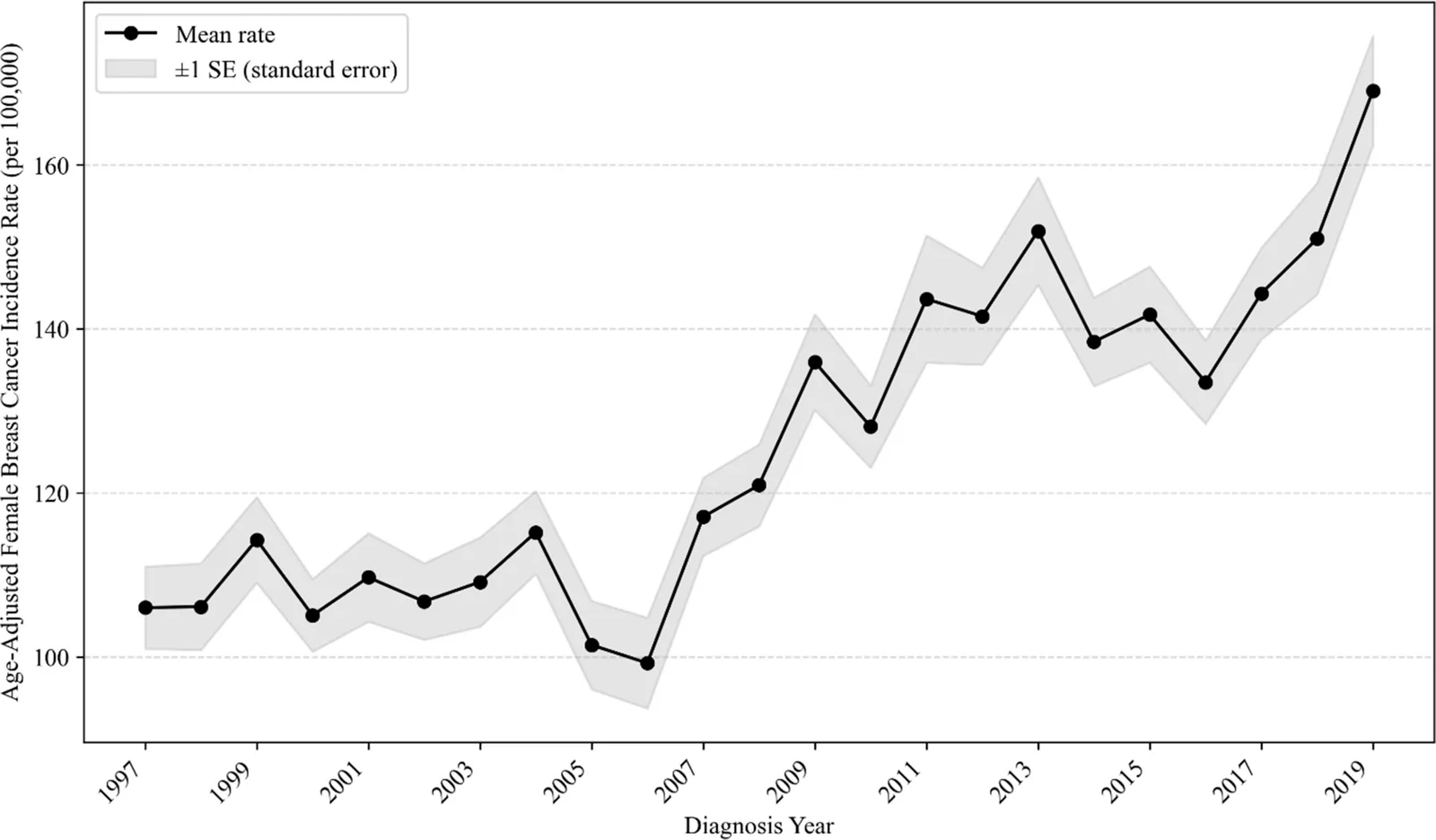
Annual mean age-adjusted female breast cancer incidence rates per 100,000 females, with ± 1 standard error (calculated as the standard deviation of census tract–level rates divided by the square root of the number of tracts contributing data in that year), by diagnosis year at the census-tract level (2010) in New Mexico, 1997–2019

During 1997 to 2019, the average age-adjusted incidence rate of FBC among census tracts in New Mexico was 133.3 cases per 100,000 females. Figure 2 shows that the mean age-adjusted female breast cancer incidence rate in New Mexico increased from 1997 (106.0 per 100,000 females) to 2019 (169.0 per 100,000 females). On average across census tracts, educational attainment varied, with 44.9% of the female population having less than some college education, 25.8% reporting some college without a degree, and 29.3% holding a college degree or higher. On average, the racial and ethnic composition of tracts was predominantly Hispanic (44.2%), followed by Non-Hispanic White (33.8%), AIAN (11.9%), Non-Hispanic Black (2.4%), and API (1.9%). The median annual female income across tracts was $21,955 (± 7,391). In terms of geography, the majority of tracts were classified as urban (69.1%), while 30.9% were rural.

After calibration, in which the effective distance cutoff was selected to maximize the significant Spearman correlation between modeled exposure intensities and monitored concentrations, the EWPM estimated exposures for the five PM_2.5_-bound metals (cobalt, chromium, manganese, mercury, and copper) were significantly positively correlated with measured concentrations from monitoring records (*p* < 0.05; Table 1). The corresponding optimal effective distances (*k*) ranged from 34 to 50 km, and the Spearman correlation coefficients ranged from 0.06 to 0.99.

**Table 1:** Calibrated parameters (effective distance *k* in EWPM) for selected chemicals

Pollutant	CAS number^a^	Spearman rank correlation test		
		Optimal *k* (km)	Coefficient^b^	*p*-value
Cobalt	7440484	50	0.999	< 0.001*
Chromium	7440473	38	0.660	0.001*
Manganese	7439965	50	0.491	0.032*
Mercury	7439976	50	0.793	0.033*
Copper	7440508	34	0.060	0.039*

^a^ CAS Registry Number: A unique identifier assigned by the Chemical Abstracts Service to each substance; order in ascending order of *p*-value

^b^Sorted by ascending Spearman rank correlation *p*-value

*Statistically significant (*p-*value < 0.05)

Table 2 shows the associations between long-term residential exposure to PM_2.5_-bound metals and age-adjusted FBC incidence rate across census tracts in New Mexico from 1997 to 2019. Adjusted rate ratios (adjusted RRs) and their corresponding 95% confidence intervals (CIs) are presented for five metals with significant associations. Compared with the unexposed group, exposure to chromium was associated with a 7% increase in FBC incidence rate (adjusted RR 1.07, 95% CI: 1.05, 1.08), while exposure to cobalt (adjusted RR 1.08, 95% CI: 1.05, 1.11) and copper (adjusted RR 1.06, 95% CI: 1.04, 1.07) showed similarly elevated risks of 8% and 6%, respectively. Manganese (adjusted RR 1.05, 95% CI: 1.02, 1.08) and mercury (adjusted RR 1.05, 95% CI: 1.03, 1.07) exposure were also significantly associated with increased FBC incidence rate.

**Table 2:** Adjusted rate ratio (adjusted RR) and 95% confidence intervals for female breast cancer incidence associated with PM_2.5_-bound metal exposures in New Mexico, 1997–2019

Chemical (CAS number)	Exposure intensity	% Census tract	Adjusted RR
Chromium (7440473)	0	10.03	1.00 (Referent) 1.07 (1.05, 1.08)*
	> 0	89.97	
Cobalt (7440484)	0	5.62	1.00 (Referent)
	> 0	94.38	1.08 (1.05, 1.11)*
Copper (7440508)	0	7.76	1.00 (Referent)
	> 0	92.24	1.06 (1.04, 1.07)*
Manganese (7439965)	0	4.42	1.00 (Referent)
	> 0	95.58	1.05 (1.02, 1.08)*
Mercury (7439976)	0	3.83	1.00 (Referent)
	> 0	96.17	1.05 (1.03, 1.07)*

^*^ Statistically significant after Bonferroni correction for multiple comparisons at level 0.05

^*^ Adjusted for female education, female racial/ethnicity population, median female income, and urbanicity of census tract

Table 3 presents the results of the age-adjusted FBC incidence rate associated with increasing levels of exposure to five PM_2.5_-bound metals. The exposure levels were divided into four groups–unexposed (reference), low, medium, and high–based on tertiles of exposure intensity greater than 0. The analysis compares the risk in low, medium, and high exposure groups relative to the unexposed (reference) group for each metal. Chromium exposure was significantly associated with increased FBC incidence rate among low and high exposure levels, with the highest risk observed in the high exposure group (adjusted RR 1.49, 95% CI: 1.23, 1.82). For cobalt, only medium and high exposure levels were significantly associated with increased rate (adjusted RR = 1.47 and 1.97, respectively), whereas low exposure was not. Copper exposure showed a pattern with low and medium exposure categories significantly associated with elevated risk, particularly the low (adjusted RR 1.55, 95% CI: 1.35, 1.78) and medium groups (adjusted RR 1.42, 95% CI: 1.23, 1.63). For manganese, only the low exposure group showed a significantly elevated adjusted RR of 1.69 (95% CI: 1.24, 2.29). In contrast, mercury exposure demonstrated significant relationships across all groups, with adjusted RRs of 1.34 (low), 1.54 (medium), and 1.47 (high). The Wald test indicated statistically significant trends for all five PM_2.5_-bound metals.

**Table 3:** Adjusted rate ratio (adjusted RR) and 95% confidence intervals for female breast cancer incidence associated with PM2.5-bound metal exposures in New Mexico, 1997–2019 with exposure intensities were divided to four categories (unexposed, low, medium, and high)

Chemical	Exposure Level	Adjusted RR	*p* value for trend
Chromium (7440473)	Reference (0)	1.00	< 0.001
	Low (0.1–13.9)	1.56 (1.30, 1.86)*	
	Medium (13.9–41.5)	1.21(1.01, 1.43)	
	High (> 41.5)	1.49 (1.23, 1.81)*	
Cobalt (7440484)	Reference (0)	1.00	< 0.001
	Low (0–40.8)	1.16 (0.92, 1.47)	
	Medium (40.9–73.5)	1.47 (1.16, 1.85)*	
	High (> 73.5)	1.97 (1.54, 2.50)*	
Copper (7440508)	Reference (0)	1.00	< 0.001
	Low (0–23.2)	1.55 (1.35, 1.78)*	
	Medium (23.3–61.6)	1.42 (1.23, 1.63)*	
	High (> 61.6)	1.19 (1.01, 1.39)	
Manganese (7439965)	Reference (0)	1.00	0.003
	Low (0–26.3)	1.69 (1.24, 2.29)*	
	Medium (26.4–145.9)	1.13 (0.79, 1.61)	
	High (> 145.9)	1.32 (0.98, 1.77)	
Mercury (7439976)	Reference (0)	1.00	< 0.001
	Low (0–0.001)	1.34 (1.09, 1.64)*	
	Medium (0.002–0.18)	1.54 (1.26, 1.88)*	
	High (> 0.18)	1.47 (1.11, 1.93)*	

^*^ Statistically significant after Bonferroni correction for multiple comparisons at level 0.05

^*^ Adjusted for female education, female racial/ethnicity population, median female income, and urbanicity of census tract

## Discussion

This study examined the relationship between long-term exposure to PM_2.5_-bound metals and age-adjusted FBC incidence rate in New Mexico from 1997 to 2019. Significant associations were observed for exposure to chromium, cobalt, copper, manganese, and mercury. These findings suggest that ambient air pollution, particularly from metal emissions, may contribute to elevated FBC incidence rate.

The findings of this study are broadly consistent with previous research linking exposure to ambient air pollution, particularly PM_2.5_-bound metals, with elevated breast cancer incidence risk. Multiple epidemiologic studies have observed associations between breast cancer and exposure to air pollutants such as traffic-related PM_2.5_, polycyclic aromatic hydrocarbons (PAHs), and heavy metals (Cheng et al. 2021; White et al. 2016). In particular, metals like chromium, cobalt, copper, and mercury have been identified as potential carcinogens or endocrine-disrupting compound (IARC 2025). For example, it has been found that women exposed to higher airborne cobalt (odds ratio [OR]: 2.0, 95% CI: 0.9, 4.4) levels had increased odds of breast cancer in Chicago between 2005 and 2008 (Kresovich et al. 2019). Similarly, elevated risks were observed in association with PM₂.₅-bound cobalt exposure in a previous study (OR:1.56, 95% CI 1.52, 1.64) in a multi-city cohort in USA, in 2011 (White et al. 2019). In addition, it has been found that the soil chromium pollution (beta = 0.01, *p* = 0.02) was associated with breast cancer hospitalization in Southeast China during 2015–2016 (Song et al. 2023b). Mercury bioaccumulation in breast cancer tissues was significantly associated with higher CXCR4 expression, suggesting a potential role for mercury in promoting tumor progression via the CXCR4–CXCL12 signaling pathway (Servadei et al. 2025). Additionally, elevated copper-to-zinc (Cu/Zn) ratios in plasma or urine have been associated with an increased risk of breast cancer development (Szwiec et al. 2024). Together, these studies provide converging evidence that exposure to various PM_2.5_-bound metals and related environmental pollutants may contribute to FBC development and progression through both environmental and biological pathways.

The biological plausibility of metal-related carcinogenesis is supported by several mechanistic pathways through which metals may contribute to breast cancer development. Many metals, such as chromium, cobalt, and mercury, are classified by the International Agency for Research on Cancer (IARC) as known or probable human carcinogens due to their ability to generate reactive oxygen species that cause oxidative DNA damage, lipid peroxidation, and impair cellular repair mechanisms (Koedrith and Seo 2011). Additionally, certain metals such as cadmium and mercury have been identified as endocrine-disrupting chemicals (EDCs) capable of mimicking estrogen and interfering with hormone signaling pathways, which are critical in breast tissue development and carcinogenesis (Diamanti-Kandarakis et al. 2009). Epigenetic modifications, such as altered DNA methylation and histone modifications induced by metal exposure, may also contribute to abnormal gene expression linked to tumor initiation and progression (Manić et al. 2022). However, because the present ecological study did not include biomarker, toxicological, or mechanistic data, these pathways should be interpreted as providing biological plausibility rather than direct mechanistic evidence from our analysis. Future studies incorporating individual-level exposure assessment, biomarkers of oxidative stress and inflammation, hormone-related markers, and molecular tumor characteristics are needed to better evaluate the biological mechanisms linking PM_2.5_-bound metal exposure to breast cancer risk.

In the categorical exposure analyses, the associations between PM_2.5_-bound metals and female breast cancer incidence did not show strictly monotonic dose–response patterns for all metals, although the trend tests were statistically significant. The adjusted RRs for some metals, particularly chromium and copper, varied across low, medium, and high exposure categories. These non-linear patterns may reflect methodological factors, including loss of information from categorizing continuous exposure estimates, skewed exposure distributions, unequal numbers of observations across exposure categories, exposure misclassification, or instability in category-specific estimates. In addition, non-linear exposure–response relationships may be biologically plausible. Metal exposures may influence carcinogenesis through mechanisms such as oxidative stress, chronic inflammation, endocrine disruption, DNA damage, and impaired DNA repair; however, these biological responses may not increase proportionally across exposure levels. Threshold effects, saturation of biological pathways, adaptive cellular responses, or competing toxic effects at higher exposure levels could also contribute to non-linear associations. Therefore, the categorical findings should be interpreted cautiously as evidence of an overall positive exposure–response trend rather than a strictly linear biological dose–response relationship.

We examined the robustness of the associations between PM_2.5_-bound metal exposures and FBC incidence rate using alternative exposure windows of 5, 8, and 12 years, in place of the 10-year average used in the primary models (Table 4). The results were largely consistent across all windows, supporting the stability of the associations. For all five metals–chromium, cobalt, copper, manganese, and mercury–statistically significant elevated adjusted RRs were observed in each time window. Notably, the estimated associations remained similar across different exposure windows, suggesting that the findings were robust to the choice of exposure averaging period. These consistent and increasingly elevated risks across varying exposure windows reinforce the reliability of the main findings and highlight the potential long-term impact of exposure to PM_2.5_-bound metals on female breast cancer risk.

**Table 4:** Adjusted rate ratio (adjusted RR) and 95% confidence intervals for female breast cancer incidence associated with PM2.5-bound metal exposures in New Mexico, using 5-, 8-, 10-, and 12-year exposure windows in New Mexico, 1999–2019

Chemical (CAS number)	Adjusted RR (10-year window)	Adjusted RR (5-year window)	Adjusted RR (8-year window)	Adjusted RR (12 -year window)
Chromium (7440473)	1.07 (1.05, 1.08)*	1.06 (1.03, 1.07)*	1.06 (1.04, 1.08)*	1.08 (1.06, 1.10)*
Cobalt (7440484)	1.08 (1.05, 1.11)*	1.06 (1.03, 1.09)*	1.07 (1.04, 1.10)*	1.11 (1.08, 1.14)*
Copper (7440508)	1.06 (1.04, 1.07)*	1.04 (1.03, 1.05)*	1.06 (1.04, 1.07)*	1.07 (1.06, 1.09)*
Manganese (7439965)	1.05 (1.02, 1.08)*	1.06 (1.03, 1.09)*	1.05 (1.02, 1.08)*	1.06 (1.03, 1.09)*
Mercury (7439976)	1.05 (1.03, 1.07)*	1.03 (1.01, 1.05)*	1.04 (1.02, 1.06)*	1.06 (1.04, 1.08)*

* Statistically significant after Bonferroni correction for multiple comparisons at level 0.05

* Adjusted for female education, female racial/ethnicity population, median female income, and urbanicity of census tract

**Fig. 3:**
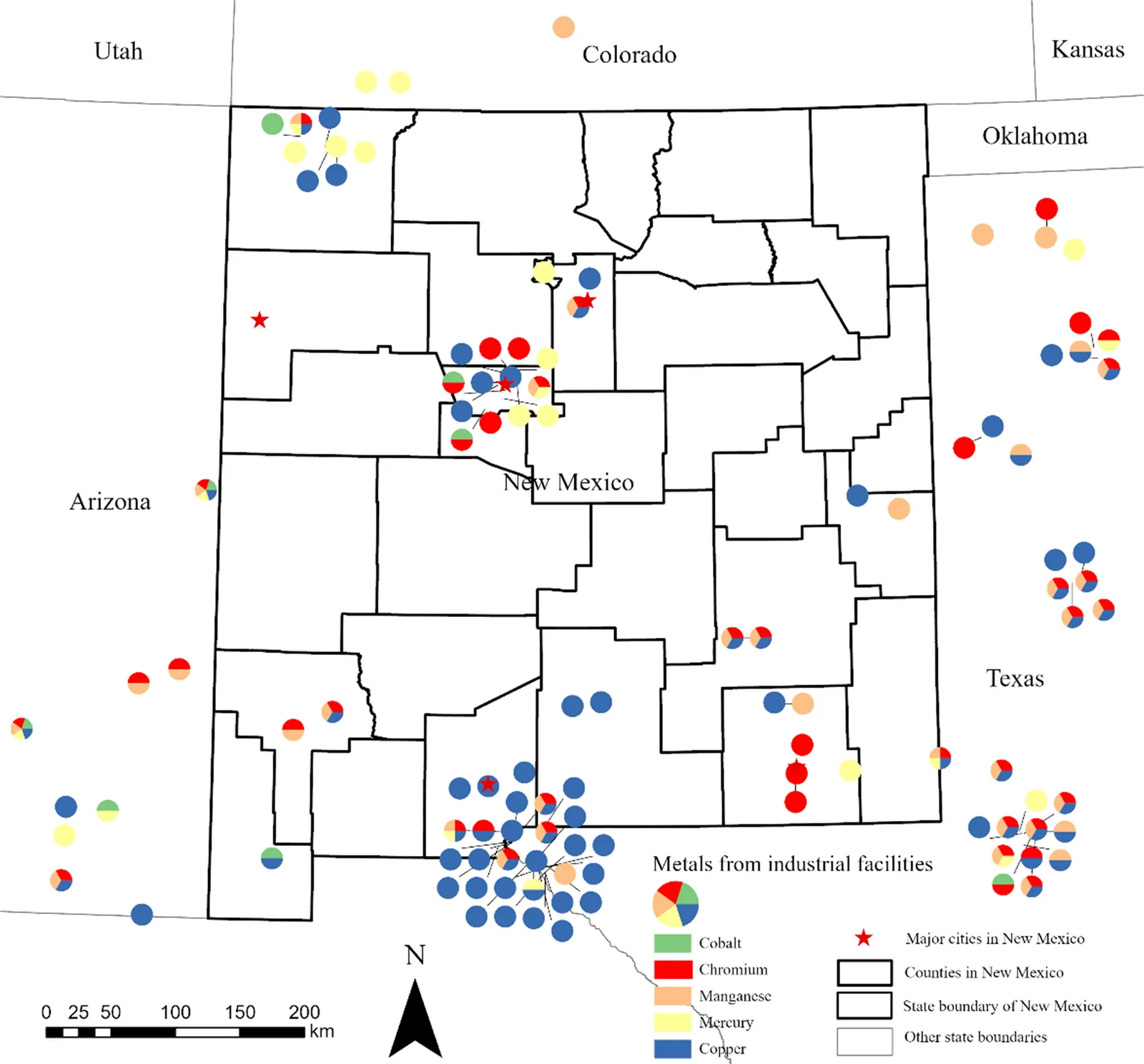
Types of metals emitted from industrial facilities in New Mexico and its surrounding area during 1987–2019

From 1987 to 2019, 119 industrial facilities in New Mexico and neighboring regions emitted five identified metals–cobalt, chromium, manganese, mercury, and copper–into the atmosphere. As shown in Fig. 3, the spatial distribution of these emissions reveals that most facilities releasing these metals were concentrated in southern, and central New Mexico, particularly around Albuquerque and the El Paso. Additional emission clusters were observed near the northwestern and southeastern New Mexico. Several facilities in western Texas also contributed to regional metal emissions. Therefore, future studies should focus more on these regions.

**Fig. 4:**
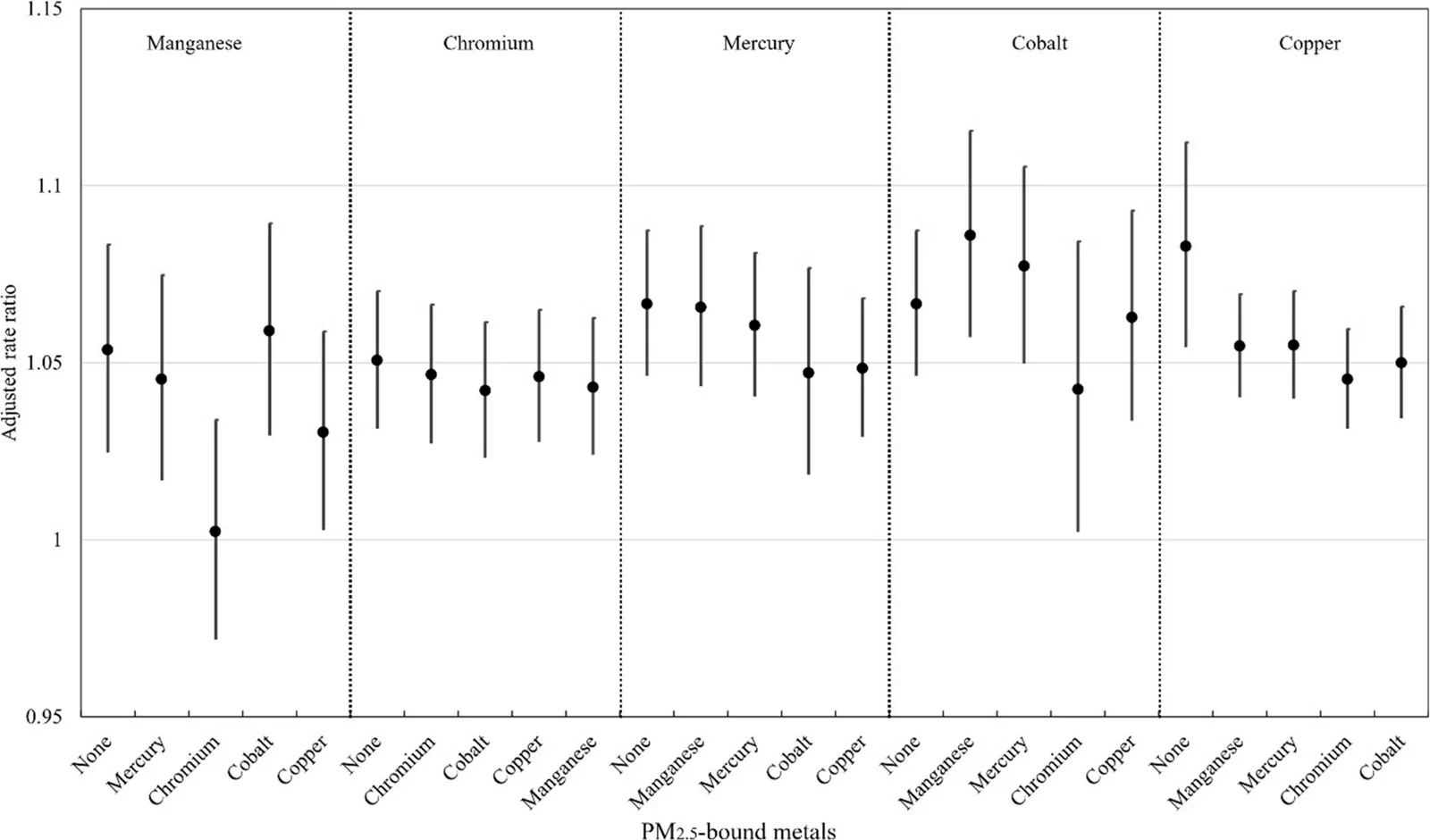
Adjusted rate ratios (adjusted RR) and 95% confidence intervals from two-pollutant models assessing the independent associations between PM_2.5_-bound metals and female breast cancer incidence in New Mexico, 1997–2019

To further assess the robustness of the observed associations and evaluate potential confounding among co-occurring pollutants, two-pollutant models were conducted by including each PM_2.5_-bound metal alongside one additional metal at a time (Tolbert et al. 2007). In these models, aRRs and 95% CIs represent the association between each metal and FBC incidence while simultaneously controlling for the concentration of another metal in the model. Results from these models showed that most metals remained statistically significant and largely consistent in magnitude, indicating that the associations were not solely driven by correlations with other metals (Fig. 4). These findings suggest that the associations for most examined PM_2.5_-bound metals were robust after adjustment for selected co-occurring metals.

This study has several limitations. First, exposure misclassification is possible, as ambient metal exposures were estimated at tract–level averages, and potential residential mobility in the years preceding diagnosis was not considered due to the unavailability of detailed residential history data. However, this approach provides a practical method for assessing exposure in large-scale, long-term studies, representing a reasonable trade-off between spatial resolution and data availability. Second, while the EWPM incorporates emission intensity and distance, it does not capture all environmental transport mechanisms, uncertainties in self-reported emissions, atmospheric transport variability, meteorological conditions, or indoor exposures that may affect actual personal exposure levels and could influence the magnitude of the observed associations. In addition, the strength of model calibration varied across metals. For example, although the calibrated EWPM for copper showed a statistically significant correlation with monitored concentrations, the correlation coefficient was relatively low, indicating weaker predictive performance compared with other metals. Therefore, exposure misclassification may be greater for metals with weaker calibration performance, particularly copper, and these findings should be interpreted with caution. Future studies using more refined exposure models, denser monitoring data, and meteorological or atmospheric dispersion information are needed to validate and improve exposure assessment. Third, potential confounding by unmeasured or residual factors could not be fully controlled due to data unavailability. These factors may include occupational exposures; lifestyle-related breast cancer risk factors such as reproductive history, hormone use, diet, physical activity, and alcohol consumption; nutritional status and physiological variability related to essential trace metals such as copper and manganese; and temporal changes in breast cancer screening, mammography uptake, and diagnostic practices. Because exposure estimates captured ambient PM_2.5_-bound metals rather than internal body burden, dietary intake, or nutritional status, findings for essential metals such as copper and manganese should be interpreted cautiously. Although the GEE approach accounted for repeated yearly observations within census tracts and calendar-year changes over the study period, data on these individual-level and temporal factors were not available; therefore, we could not fully address these factors, and residual confounding may remain. Fourth, while the analysis adjusted for key tract-level sociodemographic characteristics, the ecological study design cannot establish causal inference at the individual level and is subject to potential ecological fallacy. Therefore, associations observed at the census tract level may not necessarily reflect individual-level exposure-risk relationships. Future studies should incorporate individual-level exposure and health data, residential history, and longitudinal designs to better assess relationships between metal exposure and breast cancer risk. Fifth, age-adjusted FBC incidence rates were calculated using the 2010 census tract female population for all study years (1997–2019) to align with available tract-level covariates; this assumption of a static population structure across time may have led to under- or over-estimation of incidence rates in certain years. Sixth, breast cancer subtype based on receptor status was not included in the analysis. Specifically, ER, PR, and HER2 status were not evaluated because receptor information was not consistently available throughout the full study period. As a result, the observed associations reflect overall female breast cancer incidence and may mask differences by tumor subtype. Future studies with complete and harmonized receptor-status data are needed to evaluate whether associations between PM_2.5_-bound metals and breast cancer incidence differ by molecular subtype. Finally, the study focused only on selected metals with both emissions and monitoring data, potentially omitting other relevant pollutants or other emission sources including transportation-related emissions, mining activities, and other non-industrial contributors, which may have resulted in exposure misclassification. Future studies should include a broader range of pollutants and emission sources to improve exposure assessment.

## Conclusion

This study provides strong epidemiological evidence that long-term residential exposure to specific PM_2.5_-bound metals, including chromium, cobalt, copper, manganese, and mercury, is associated with an increased age-adjusted FBC incidence rate in New Mexico between 1997 and 2019. When exposure was categorized into unexposed, low, medium, and high levels, a significant increasing trend was observed for all five metals. The associations remained robust across multiple exposure windows and the results of two-pollutant models also suggest independent effects of individual metals. The findings underscore the importance of addressing air-borne metal components exposures as part of breast cancer prevention and control strategies. Future research should aim to validate these associations using longitudinal study, finer spatial exposure models, and biological markers of exposure and effect. Enhanced surveillance and regulatory efforts targeting airborne toxic metals could help reduce environmental risk burdens and contribute to more equitable cancer prevention.

## Data Availability

The datasets used during this study are not publicly available.
